# A surveillance and control of Ebola Outbreak Disease at Télimélé, Guinea Conakry 2014

**DOI:** 10.1186/2047-2994-4-S1-P3

**Published:** 2015-06-16

**Authors:** JMV Namahoro, U Hogan

**Affiliations:** 1 Infection Prevention and Control, Stellenbosch University, Cape Town, South Africa

## Introduction

The Global Outbreak Alert and Response Network (GOARN) have been involved in infectious diseases outbreak response low-resource settings for many years. The current Ebola Virus Disease (EVD) outbreak in West Africa, originated in Conakry Guinea. Burial rituals, traditional healer practices, illegal healthcare practices, ineffective contact tracing of exposed persons and a weak healthcare infrastructure are major contributing factors to the ongoing Ebola transmission in this setting. The purpose of this study was to describe a successful attempt to stop the EVD outbreak through early, systematic isolation of cases.

## Objectives

To identify contacts of ill or deceased persons and monitor these individuals daily for the duration of the 21 day incubation period.

## Methods

A descriptive study design was used with data collected on a contact tracing/monitoring audit form. Two criteria were considered when attempting to identify possible EVD contacts: the immediate neighborhood of the affected individual and a history of participation in burial rites. All exposed individuals were monitored for 21 days from the date of exposure.

## Results

A total of 118 exposed persons were observed for 21 days. Of this cohort, 21% (n=25) developed EVD, and 36% (n=9) of these individuals died. Women were more likely to develop infection than the men (57% vs. 43%) and adults were affected more frequently than children. Preparations for burial and the associated funeral rites were a major transmission risk factor among these cases.

## Conclusion

The current West African outbreak as a whole can be stopped in two months. Ebola virus is transmitted among humans through close and direct physical contact with infected bodily fluids. Early identification, systematic isolation of cases, standard precautions, improved community awareness about risk factors for Ebola transmission, individual protective measures and safe burial practices are the key methods to prevent EVD.

## Disclosure of interest

None declared.

